# Salidroside Inhibits HMGB1 Acetylation and Release through Upregulation of SirT1 during Inflammation

**DOI:** 10.1155/2017/9821543

**Published:** 2017-12-03

**Authors:** Zhilin Qi, Yao Zhang, Shimei Qi, Liefeng Ling, Lin Gui, Liang Yan, Jun Lv, Qiang Li

**Affiliations:** ^1^Department of Biochemistry, Wannan Medical College, Wuhu, Anhui, China; ^2^Anhui Province Key Laboratory of Active Biological Macromolecules, Wuhu, Anhui, China; ^3^Department of Microbiology and Immunology, Wannan Medical College, Wuhu, Anhui, China

## Abstract

HMGB1, a highly conserved nonhistone DNA-binding protein, plays an important role in inflammatory diseases. Once released to the extracellular space, HMGB1 acts as a proinflammatory cytokine that triggers inflammatory reaction. Our previous study showed that salidroside exerts anti-inflammatory effect via inhibiting the JAK2-STAT3 signalling pathway. However, whether salidroside inhibits the release of HMGB1 is still unclear. In this study, we aim to study the effects of salidroside on HMGB1 release and then investigate the potential molecular mechanisms. In an experimental rat model of sepsis caused by CLP, salidroside administration significantly attenuated lung injury and reduced the serum HMGB1 level. In RAW264.7 cells, we investigated the effects of salidroside on LPS-induced HMGB1 release and then explored the underlying molecular mechanisms. We found that salidroside significantly inhibited LPS-induced HMGB1 release, and the inhibitory effect was correlated with the HMGB1 acetylation levels. Mechanismly, salidroside inhibits HMGB1 acetylation through the AMPK-SirT1 pathway. In addition, SirT1 overexpression attenuated LPS-induced HMGB1 acetylation and nucleocytoplasmic translocation. Furthermore, in SirT1 shRNA plasmid-transfected cells, salidroside treatment enhanced SirT1 expression and reduced LPS-activated HMGB1 acetylation and nucleocytoplasmic translocation. Collectively, these results demonstrated that salidroside might reduce HMGB1 release through the AMPK-SirT1 signalling pathway and suppress HMGB1 acetylation and nucleocytoplasmic translocation.

## 1. Introduction

High-mobility group box 1 (HMGB1), a ubiquitous nonhistone DNA-binding protein, is constitutively expressed in the nucleus of eukaryotic cells and plays important roles in maintaining the nucleosome structure and stability, chromatin remodelling, and regulation of gene transcription [1–3]. In addition to these intracellular functions, HMGB1 can be released to the extracellular space and acts as a proinflammatory cytokine to induce an inflammatory reaction [4]. To date, we have learned that HMGB1 can be either actively released by activated immune cells or passively released from damaged and necrotic cells [5, 6], and nucleocytoplasmic translocation is required for the active release pathway of HMGB1 [7]. Once released into the extracellular space, HMGB1 binds with its receptors including TLR2 (toll-like receptor 2), TLR4, and RAGE (receptor for advanced glycation end products) and then participates in a number of signalling pathways [8]. Growing evidences suggest that HMGB1 plays an important role in inflammatory diseases such as sepsis [9, 10]. Therefore, targeting HMGB1 may be an important strategy for the treatment of sepsis and other inflammatory diseases.

Several posttranslational modifications, such as phosphorylation, acetylation, and methylation, are involved in the translocation of HMGB1 from the nucleus to the cytoplasm [7, 11, 12]. A recent study showed that HMGB1 is acetylated and released from RAW264.7 cells upon LPS stimulation [13]. It has also been reported that an NAD-dependent class III histone deacetylase (HDAC) plays an important role in regulating acetylated HMGB1 release from neurons in response to ethanol exposure [14]. As an NAD-dependent class III histone deacetylase, sirtuin 1 (SirT1) was demonstrated to regulate HMGB1 hyperacetylation and extracellular release [15]. AMP-activated protein kinase (AMPK), a conserved serine/threonine kinase, is one of the main regulators of whole-body energy homeostasis [16]. SirT1 also acts as an energy sensor [17]; SirT1 and AMPK cooperate to regulate metabolic pathways. A recent study reported that AMPK activates SirT1 by increasing the expression of nicotinamide phosphoribosyltransferase (Nampt) [18]. In addition, emerging evidence suggested that the AMPK-SirT1 signalling pathway also regulates inflammatory signalling in various cells [19, 20].

**Figure 1:**
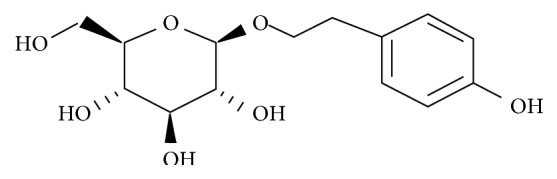
The chemical structure of salidroside.

Salidroside (SAL, p-hydroxyphenethyl-*β*-D-glucoside, structure shown in Figure 1) is an active component isolated from *Rhodiola rosea* [21]. It has been reported to exert various pharmacological properties including anti-inflammation [21], antitumour [22, 23], and neuroprotection [24]. Our previous study showed that salidroside suppressed the release of inflammatory cytokines and mediators induced by LPS in RAW264.7 cells and peritoneal macrophages by inhibiting the JAK2-STAT3 signalling pathway [25]. However, whether salidroside inhibits the release of HMGB1 and the potential molecular mechanisms is still unclear.

In the present study, our aim was to investigate the effects of salidroside on HMGB1 release and explore whether the effect was associated with AMPK-SirT1-mediated HMGB1 acetylation.

## 2. Materials and Methods

### 2.1. Reagents and Antibodies

Salidroside (>98% purity) and LPS were purchased from Sigma. The primary antibodies against GAPDH, *β*-actin, lamin B1, TBP, SirT1, and phospho-AMPK (Thr172) were the products of Cell Signaling Technology (CST). The anti-HMGB1, anti-phosphoserine, and anti-acetyl lysine antibodies were all purchased from Abcam. All of the secondary antibodies used for Western blotting were purchased from LI-COR Biotechnology. Precleared protein A/G plus agarose beads used in coimmunoprecipitation (IP) was obtained from Santa Cruz Biotechnology Inc.

### 2.2. Cell Culture and Passage

RAW264.7 cells, obtained from Kunming Cell Bank (Kunming, China), were cultured in Dulbecco's modified Eagle medium (DMEM) containing 10% foetal bovine serum, 100 *μ*g/ml of streptomycin, and 100 U/ml penicillin at 37°C and 5% CO_2_. The cells were passaged every 1-2 days.

### 2.3. Measuring HMGB1 Levels

The amounts of HMGB1 in the cell culture supernatants were detected using a double-antibody sandwich ELISA kit purchased from Wuhan Huamei Biotech Co. Ltd. (Wuhan, China). Operations were carried out according to the protocol provided by the manufacturer.

### 2.4. Plasmids and Transfection

Overexpression and negative control plasmids of SirT1 were presented by Professor Zhihao Wu (Department of Biology, Wannan Medical College). The SirT1 shRNA and negative control plasmids were purchased from Shanghai Genechem Co. Ltd. (Shanghai, China). Cells were planted in 6-well plates for 24 h and then transfected with the Lipofectamine 3000 transfection reagent (Invitrogen) according to the manufacturer's protocol. The transfected cells were treated for the indicated times and subjected to Western blotting or coimmunoprecipitation.

### 2.5. Western Blotting and Coimmunoprecipitation

Cells were washed with ice-cold PBS, lysed on ice for 30 min in cell lysis buffer (Beyotime Biotechnology, China), and combined with the protease inhibitor cocktail (Roche Applied Science, Indianapolis, IN, USA). Lysates were collected and centrifuged (12,500 rpm) at 4°C for 15 min. Equal amounts of protein were immunoprecipitated with the indicated antibody at 4°C overnight. Then, 20 *μ*l of precleared protein A/G plus agarose beads was added and incubated with the immunocomplexes for additional 2 h at 4°C. After washing with cold lysis buffer three times, the immunoprecipitates were separated by SDS-PAGE and then transferred onto nitrocellulose membranes (Millipore, USA); the membranes were blocked with 5% skim milk for 1 h at room temperature. After washing with TBST, the membranes were incubated with the indicated primary antibody overnight at 4°C and then washed with TBST three times. After washing, the membranes were incubated with an IRDye800 fluorophore-conjugated secondary antibody for 1 h at room temperature in the dark. The blots were analysed by the LI-COR Odyssey Infrared Imaging System (LICOR Biosciences, Lincoln, NE), and proteins were quantified by the LI-COR Odyssey analysis software.

### 2.6. Nuclear and Cytoplasmic Protein Extraction

Briefly, after treatment, the RAW264.7 cells were lysed and the nuclear and cytoplasmic proteins were separated and collected using a nucleocytoplasmic separation kit (Beyotime Biotechnology, China). Operations were strictly carried out according to the manufacturer's instructions.

### 2.7. Laser Confocal Experiment

RAW264.7 or transfected RAW264.7 cells were seeded in small confocal laser dishes; on the next day, the cells were pretreated with salidroside for 2 h and then stimulated with LPS for the indicated time points. Cells were fixed with 4% polyformaldehyde, permeabilized with 0.2% Triton X-100, and blocked with 3% BSA. After washing in PBS, the cells were incubated with an HMGB1 primary antibody overnight at 4°C; then, a goat anti-rabbit IgG Alexa Fluor 555-conjugated secondary fluorescent antibody was incubated for 1 h at room temperature in the dark. Nuclei were stained with DAPI for 3 min. Images were captured using a LEICA TCS SP8 microscope.

### 2.8. Animal Model of Sepsis

This study was approved by the Animal Care and Use Committee of Wannan Medical College. Animals were handled in accordance with the requirements of the Provisions and General Recommendation of Chinese Experimental Animals Administration Legislation. Briefly, sepsis was induced in Wistar rats weighing 180–220 g by caecal ligation and puncture (CLP) as previously described [26]. All rats were anaesthetized with sodium pentobarbital (30 mg/kg) and divided into three groups randomly before surgery: the control group (sham operation group), CLP group, and salidroside (SAL) group. In the control group, rats were anaesthetized and underwent surgery without CLP. In the CLP group, rats were anaesthetized and subjected to CLP. In the SAL group, salidroside (20 mg/kg) was administered to the rats, and they were subjected to CLP after 30 min. Blood was collected 12 h after the CLP to detect the serum levels of HMGB1. In parallel experiments, 12 h after CLP, lung tissue was extracted from rats euthanized under anaesthesia.

### 2.9. Histological Examination

Lung tissue was isolated from rats, and was then washed with PBS, fixed with formalin, and embedded in paraffin. Finally, the sections were stained with haematoxylin and eosin (H&E).

### 2.10. Statistical Analysis

All data were expressed as the mean ± SD. Statistical analysis was performed using SPSS software (SPSS, version 17.0; SPSS Inc., Chicago, IL, USA). The significance of differences was calculated with one-way analysis of variance (ANOVA). *P* < 0.05 was considered to indicate a significant difference.

## 3. Results

### 3.1. Salidroside Decreases the Serum HMGB1 Level and Improves Lung Injury in CLP Rats

**Figure 2:**
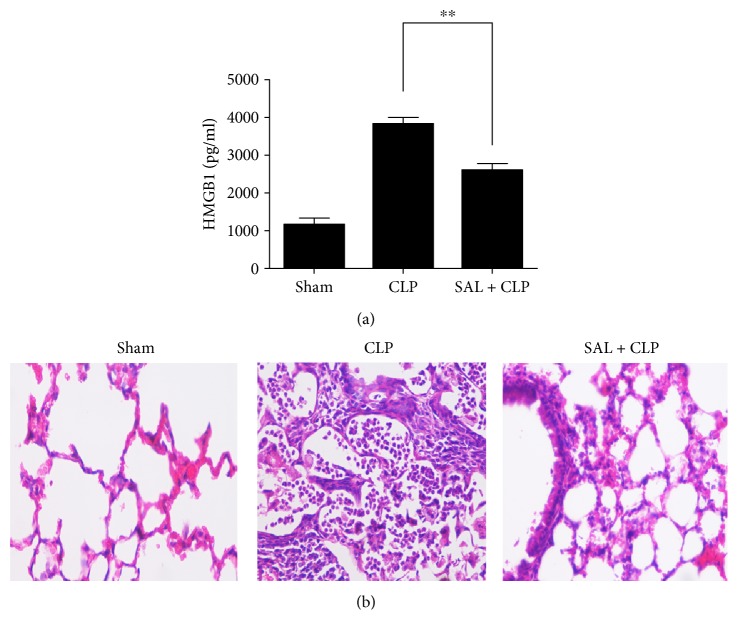
Salidroside decreases serum HMGB1 level and improves the lung injury in CLP rats. Salidroside was administrated to the rats, and rats were subjected to CLP after 30 min. (a) Blood was collected after CLP, and serum HMGB1 level was determined by ELISA (*n* = 5 rats/group; ^∗∗^*P* < 0.01). Data were shown as mean ± SD. (b) Lung tissues from different groups of rats were collected and subjected to H&E staining and then examined by light microscopy.

Our previous study found that salidroside attenuated serum IL-6 and TNF-*α* levels in LPS-induced acute lung injury [25]. In the present study, to further certify the anti-inflammatory role of salidroside, we wanted to evaluate the effect of salidroside on HMGB1 release and lung injury in a CLP-induced septic model.  Figure 2(a) reveals that the level of serum HMGB1 in the salidroside group at 12 h after CLP was obviously reduced compared with that in the CLP group. Because lung tissue is particularly susceptible to acute injury in sepsis [10], we performed histological examinations to evaluate the effect of salidroside on CLP-induced lung injury, which is characterized by congestion, oedema, and inflammatory cell infiltration. Our results showed that histological damage, oedema, and inflammatory cell infiltration were all clearly alleviated in the salidroside group compared with the CLP group (Figure 2(b)).

### 3.2. Salidroside Attenuates LPS-Induced HMGB1 Release in RAW264.7 Cells

**Figure 3:**
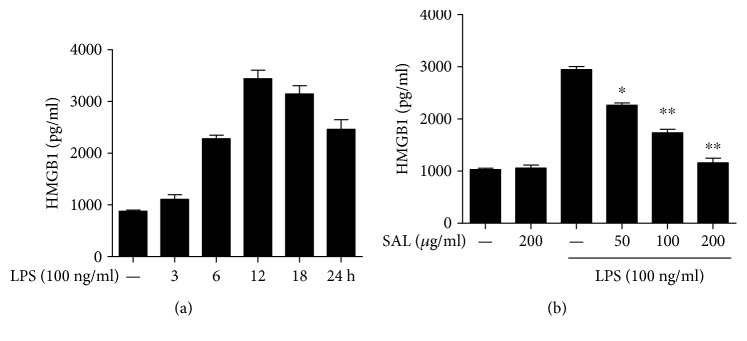
Salidroside attenuates LPS-induced HMGB1 release in RAW264.7 cells. (a) Cells were treated with LPS for different time points. (b) Cells were pretreated with salidroside for 2 h and then stimulated with LPS for 12 h; the levels of HMGB1 in the cell culture supernatants were detected by ELISA. ^∗^*P* < 0.05, ^∗∗^*P* < 0.01 versus control group.

To clarify whether the reduced serum HMGB1 in salidroside-treated CLP rats is because of the inhibitory effect of salidroside on HMGB1 release, we selected RAW264.7 cells to detect the effect of salidroside on LPS-induced HMGB1 release. Because our previous study showed that salidroside had no cytotoxicity on cell viability even at a high dose of 400 *μ*g/ml [25]. We stimulated the cells with 50–200 *μ*g/ml of salidroside in this study. We first detected the release of HMGB1 upon LPS stimulation in RAW264.7 cells. Cells were treated with 100 ng/ml of LPS for different time points (3, 6, 12, 18, and 24 h); the levels of HMGB1 in culture supernatants were determined using an ELISA kit.  Figure 3(a) shows that LPS induced HMGB1 release and that the level of HMGB1 peaked at approximately 12 h. Second, we investigated the effects of salidroside on LPS-activated HMGB1 release. RAW264.7 cells were pretreated with different doses of salidroside (50, 100, and 200 *μ*g/ml) for 2 h and then treated with 100 ng/ml of LPS for 12 h. The results in Figure 3(b) show that salidroside obviously dose-dependently reduced HMGB1 release.

### 3.3. Salidroside Inhibits LPS-Induced HMGB1 Nucleocytoplasmic Translocation in RAW264.7 Cells

**Figure 4:**
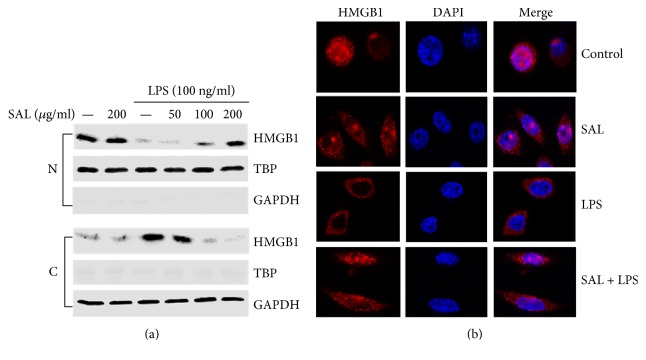
Salidroside inhibits the translocation of HMGB1 from the nucleus to the cytoplasm induced by LPS in RAW264.7 cells. Cells were pretreated with salidroside for 2 h and then treated with LPS for 12 h. (a) Nuclear and cytoplasmic proteins were extracted, respectively, and Western blotting was used to determine the amounts of nuclear and cytoplasmic HMGB1. (b) After treatment, the cells in laser confocal dishes were fixed with 4% paraformaldehyde for 30 min, blocked with 3% BSA for 1 h, incubated with an anti-HMGB1 antibody overnight, and then incubated with an Alexa Fluor 555 goat anti-rabbit IgG (red) antibody for 1 h in the dark. Cell nuclei were stained with DAPI (blue) for 3 min, and the localization of HMGB1 in cells was visualized by confocal microscopy.

Nucleocytoplasmic translocation of HMGB1 is a key step for HMGB1 release during inflammation. To examine the effect of salidroside on HMGB1 nucleocytoplasmic translocation, the amounts of HMGB1 in nuclear and cytoplasmic proteins were detected by nucleocytoplasmic separation. The localization of HMGB1 in the nucleus and cytoplasm was determined by a laser confocal microscope. RAW264.7 cells were pretreated with salidroside (200 *μ*g/ml) for 2 h and then stimulated with LPS (100 ng/ml) for 12 h. The cytoplasmic and nuclear proteins were collected, respectively. The results in Figure 4(a) show that the protein level of nuclear HMGB1 was reduced after stimulation with LPS and pretreatment with salidroside clearly suppressed the response. At the same time, the expression of cytoplasmic HMGB1 showed the opposite changes. The results of laser confocal microscopy reveal that HMGB1 (red) is mainly localized to the nucleus (blue) in the control group; after the treatment with LPS, nuclear HMGB1 was transferred to the cytoplasm and this translocation of HMGB1 was inhibited in the cells pretreated with salidroside (Figure 4(b)). Our results suggest that salidroside inhibits the nucleocytoplasmic translocation of HMGB1 induced by LPS in RAW264.7 cells.

### 3.4. Salidroside Reduces the Acetylation but Not the Phosphorylation of HMGB1 Induced by LPS in RAW264.7 Cells

**Figure 5:**
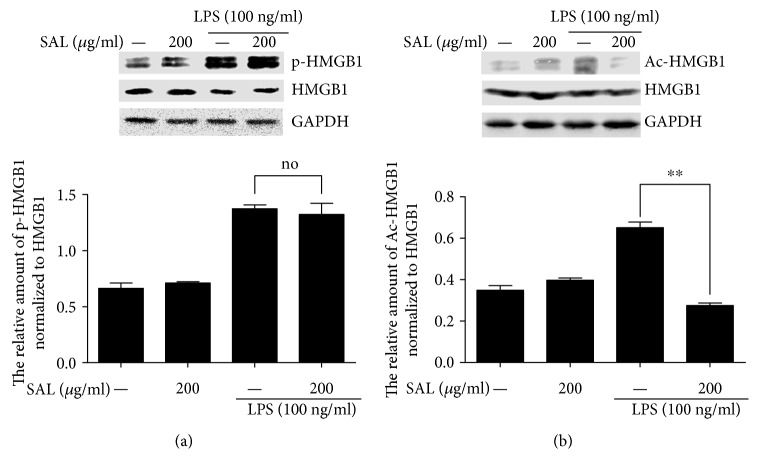
Salidroside attenuates the acetylation but not phosphorylation of HMGB1 induced by LPS in RAW264.7 cells. The cells were preincubated with salidroside for 2 h and then stimulated with LPS for 4 h, and whole-cell lysates were immunoprecipitated with an anti-HMGB1 antibody. (a) Phosphorylated HMGB1 was detected by an anti-phosphoserine antibody. (b) The acetylation of HMGB1 was analysed by an anti-acetyl lysine antibody through Western blotting. The histogram showed the relative expression of phosphorylated HMGB1 or acetylated HMGB1 normalized to total HMGB1. The experiments were done in triplicate, and data were shown as mean ± SD. ^∗∗^*P* < 0.01 compared with the group stimulated with LPS.

Because many posttranslational modifications, including phosphorylation and acetylation, for example, are involved in the nucleocytoplasmic translocation of HMGB1, we studied the effects of salidroside on LPS-induced HMGB1 phosphorylation and acetylation in RAW264.7 cells. Cells were preincubated with salidroside (200 *μ*g/ml) for 2 h and then treated with 100 ng/ml of LPS for 4 h. Total proteins were extracted, and then, IP was used to determine the phosphorylation and acetylation of HMGB1. As shown in Figure 5(a), HMGB1 was significantly phosphorylated upon LPS stimulation. However, upon pretreatment with salidroside, the level of HMGB1 phosphorylation was not affected. The acetylation of HMGB1 was also detected. Our results suggest that LPS treatment induced the acetylation of HMGB1 in RAW264.7 cells and that pretreatment with salidroside could inhibit LPS-induced HMGB1 acetylation distinctly (Figure 5(b)). Taken together, these results show that salidroside reduced LPS-activated HMGB1 acetylation but not phosphorylation in RAW264.7 cells.

### 3.5. Salidroside Upregulates the Expression of SirT1 through AMPK Signalling in RAW264.7 Cells

**Figure 6:**
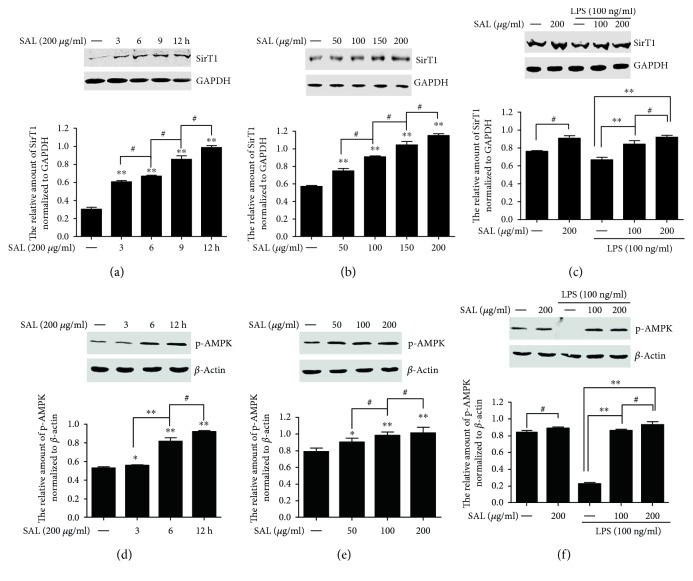
Salidroside upregulates the expression of SirT1 via AMPK signalling in RAW264.7 cells. (a, d) The cells were treated with salidroside for different time points. (b, e) Cells were treated with different concentrations of salidroside for the indicated time. (c, f) Cells were pretreated with salidroside for 2 h and then treated with LPS for 12 h. Total protein was collected, and then, the amounts of SirT1 and AMPK phosphorylation were detected by Western blotting. Equal loading protein was confirmed by immunoblotting with a GAPDH or *β*-actin antibody. The experiments were repeated three times, and data were shown as mean ± SD. ^∗^*P* < 0.05, ^∗∗^*P* < 0.01 compared with the control group (a, b, d, and e) or LPS group (c and f), ^#^*P* < 0.05 comparison between the two groups.

SirT1, a member of the NAD-dependent class III histone deacetylases, was found to affect the acetylation and release of HMGB1. We further explored whether salidroside affected the expression of SirT1 in the present study. RAW264.7 cells were treated with salidroside (200 *μ*g/ml) for different time points, or different concentrations of salidroside (50, 100, and 200 *μ*g/ml) for 12 h, or pretreated with salidroside (200 *μ*g/ml) for 2 h and then treated with LPS (100 ng/ml) for additional 12 h. Total proteins were extracted, and the levels of SirT1 were determined by Western blotting. Figures 6(a) and 6(b) show that salidroside enhanced the expression of SirT1 in a time- and dose-dependent manner. In addition, LPS stimulation reduced the level of SirT1; however, salidroside pretreatment dose-dependently reversed the reduction of SirT1 induced by LPS (Figure 6(c)). It has been reported that the AMPK-SirT1 signalling pathway regulates inflammatory signalling in various cells and that AMPK can activate SirT1. Thus, we next investigated whether salidroside upregulated SirT1 expression via AMPK signalling. RAW264.7 cells were treated with salidroside (200 *μ*g/ml) for different time points, or different concentrations of salidroside (50, 100, and 200 *μ*g/ml) for 12 h, or salidroside (200 *μ*g/ml) plus LPS (100 ng/ml) for 12 h. Total proteins were extracted, and the levels of phosphorylated AMPK were determined by Western blotting. The results show that phosphorylation of AMPK was enhanced upon salidroside stimulation in a time- and dose-dependent manner (Figures 6(d) and 6(e)). LPS stimulation obviously inhibited AMPK phosphorylation, and salidroside treatment could significantly reverse the inhibition of LPS on AMPK phosphorylation (Figure 6(f)). These results suggest that salidroside might upregulate SirT1 via AMPK signalling.

### 3.6. SirT1 Overexpression Inhibits the Acetylation and Nucleocytoplasmic Translocation of HMGB1 Induced by LPS in RAW264.7 Cells

**Figure 7:**
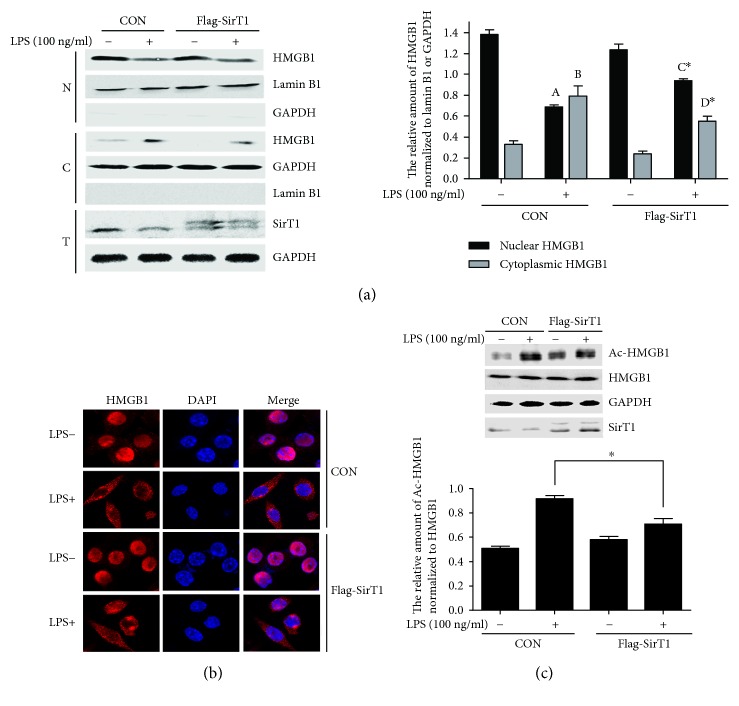
SirT1 overexpression inhibits the acetylation and translocation of HMGB1 induced by LPS in RAW264.7 cells. The cells were transfected with Flag-SirT1 and control plasmids; after 24 h transfection, the cells were treated with LPS for 12 h. (a) Nuclear and cytoplasmic and total proteins were extracted, respectively; the levels of HMGB1 were detected by Western blotting. The histogram showed the relative expression of HMGB1 in nuclear and cytoplasmic protein normalized to lamin B1 or GAPDH, respectively. The experiments were done in triplicate, and data were shown as mean ± SD. ^A,B^*P* < 0.01 compared with the group stimulated without LPS in control plasmid-transfected cells. ^C,D^*P* < 0.01 compared with the group stimulated without LPS in Flag-SirT1-transfected cells. ^∗^*P* < 0.05compared with the control plasmid-transfected and LPS-treated cells. (b) After transfection and LPS treatment, the cells in laser confocal dishes were fixed with 4% paraformaldehyde, blocked with 3% BSA, incubated with an anti-HMGB1 antibody, and then incubated with an Alexa Fluor 555 goat anti-rabbit IgG (red) antibody. Cell nuclei were stained with DAPI (blue), and the localization of HMGB1 was visualized by confocal microscopy. (c) The cells were transfected with control and Flag-SirT1 plasmids for 24 h, then stimulated with LPS for 4 h; HMGB1 acetylation was determined by IP. The histogram showed the relative expression of acetylated HMGB1 normalized to total HMGB1. The experiments were done in triplicate, and data were shown as mean ± SD. ^∗^*P* < 0.05 compared with the control plasmid-transfected group stimulated with LPS.

Our results presented above indicate that salidroside reduces the acetylation and nucleocytoplasmic translocation of LPS-induced HMGB1 by upregulating SirT1 expression. To further verify the role of SirT1 in the regulation of HMGB1 acetylation and nucleocytoplasmic translocation, we overexpressed SirT1 plasmids and negative control plasmids in RAW264.7 cells. Twenty-four hours after transfection, the cells were treated with or without LPS (100 ng/ml) for the indicated time points. The distribution of HMGB1 was detected by nucleocytoplasmic separation and confocal experiments, the acetylation of HMGB1 was detected by IP, and the efficiency of transfection of SirT1 was analysed by grey-level scanning. Compared with the control plasmids, the SirT1 overexpression efficiencies were 140.8 and 152.40%, respectively. The results obtained from Figure 7(a) show that for the cells transfected with control plasmids, after LPS treatment, the level of HMGB1 in the nucleus was clearly attenuated compared to that for the cells treated without LPS. Contrarily, the expression of HMGB1 protein in the cytoplasm presented the opposite change. However, the changes of HMGB1 levels in the nucleus and cytoplasm were suppressed significantly in SirT1 plasmid-transfected cells compared with the control plasmid-transfected cells. The distribution of HMGB1 was determined by laser confocal microscopy. In Figure 7(b), we show that, in control plasmid-transfected RAW264.7 cells, HMGB1 (red) is mainly located in the nucleus (blue), but after LPS stimulation, HMGB1 is clearly translocated from the nucleus to the cytoplasm. In the cells transfected with a SirT1 overexpression plasmid, the translocation of HMGB1 induced by LPS was clearly attenuated compared with that in the control plasmid-transfected group. We also investigated the effects of SirT1 overexpression on LPS-activated HMGB1 acetylation. The results in Figure 7(c) indicate that, in the cells transfected with control plasmids, LPS treatment induced the acetylation of HMGB1 obviously, but the enhanced HMGB1 acetylation in the control plasmid-transfected group was significantly reduced in the SirT1 plasmid-transfected cells. In other words, our results show that SirT1 overexpression can inhibit LPS-induced HMGB1 acetylation and nucleocytoplasmic translocation in RAW264.7 cells.

### 3.7. Salidroside Reverses LPS-Induced HMGB1 Acetylation and Nucleocytoplasmic Translocation in SirT1 shRNA-Transfected RAW264.7 Cells

**Figure 8:**
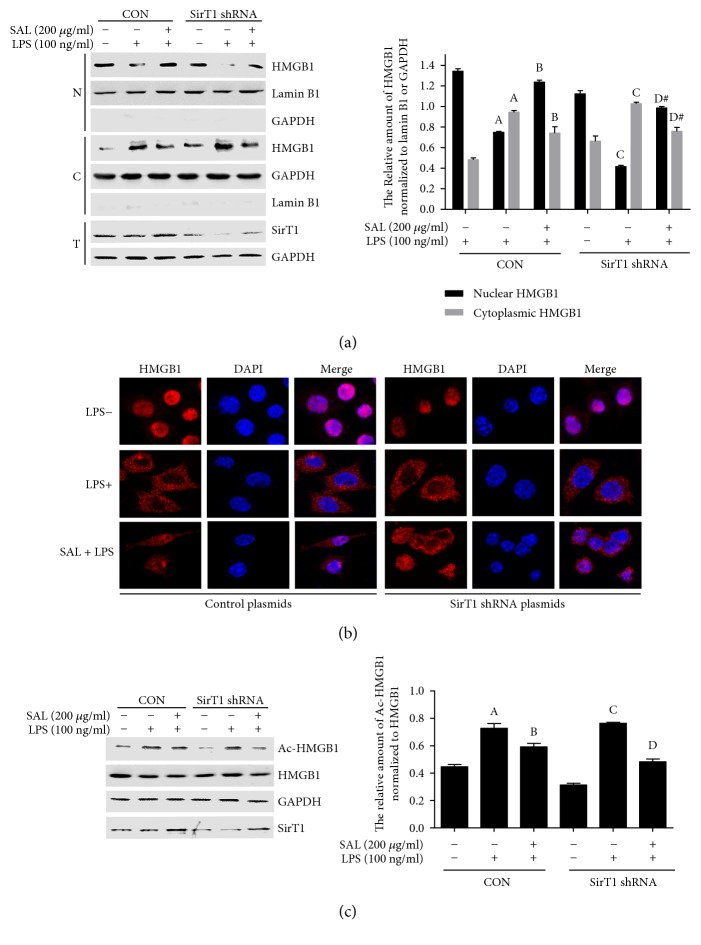
Salidroside reverses LPS-induced HMGB1 acetylation and nucleocytoplasmic translocation in SirT1 shRNA-transfected RAW264.7 cells. The cells were transfected with SirT1shRNA and control plasmids for 48 h; after the transfection, cells were pretreated with salidroside for 2 h and then treated with LPS for 12 h. (a) Nuclear and cytoplasmic and total protein were extracted, respectively; the levels of HMGB1 were detected by Western blotting. The histogram showed the relative expression of HMGB1 in nuclear and cytoplasmic protein normalized to lamin B1 or GAPDH, respectively. The experiments were done three times, and data were shown as mean ± SD. ^A^*P* < 0.01 compared with the group stimulated without LPS in control plasmid-transfected cells. ^B^*P* < 0.01 compared with the group stimulated with LPS in control plasmid-transfected cells. ^C^*P* < 0.01 compared with the group stimulated without LPS in SirT1 shRNA-transfected cells. ^D^*P* < 0.01 compared with the group stimulated with LPS in SirT1 shRNA-transfected cells. ^#^*P* < 0.01 compared with the group stimulated with LPS in SirT1 shRNA-transfected cells. The localization of HMGB1 was visualized by confocal microscopy. (b) The cells were transfected with control plasmids and SirT1 shRNA plasmids; after 48 h transfection, cells were pretreated with salidroside for 2 h and then stimulated with LPS for another 4 h. HMGB1 acetylation was determined by IP. (c) The histogram showed the relative expression of acetylated HMGB1 normalized to total HMGB1. The experiments were done in triplicate, and data were shown as mean ± SD. ^A^*P* < 0.01 compared with the group stimulated without LPS in control plasmid-transfected cells. ^B^*P* < 0.01 compared with the group stimulated with LPS in control plasmid-transfected cells. ^C^*P* < 0.01 compared with the group stimulated without LPS in SirT1 shRNA-transfected cells. ^D^*P* < 0.01 compared with the group stimulated with LPS in SirT1 shRNA-transfected cells.

To further verify the roles of salidroside and SirT1 in HMGB1 acetylation and nucleocytoplasmic translocation, we transfected SirT1 shRNA and negative control plasmids into RAW264.7 cells. Forty-eight hours after transfection, the cells were treated with either LPS or salidroside plus LPS for 12 h. The expression of HMGB1 in nuclear and cytoplasmic proteins was detected by Western blotting, the location of HMGB1 was determined by confocal microscopy, the acetylation of HMGB1 was detected by IP, and the transfection efficiency of SirT1 was analysed by grey-level scanning. As shown in Figure 8(a), compared with the control plasmids, the SirT1 interference efficiencies were 59.34, 63.50, and 55.63%, respectively. In the SirT1 shRNA-transfected cells, LPS induced the clear translocation of HMGB1 from the nucleus to the cytoplasm. However, the translocation of HMGB1 in SirT1 shRNA-transfected cells was reversed following pretreatment with salidroside. Through detection of SirT1 expression in the total protein, we found that salidroside rescued the reduction of SirT1 induced by SirT1 shRNA (Figure 8(a)). The localization of HMGB1 detected by confocal microscopy also showed that salidroside could reverse LPS-induced HMGB1 nucleocytoplasmic translocation in SirT1 shRNA-transfected RAW264.7 cells (Figure 8(b)). To further verify the effect of salidroside on LPS-activated HMGB1 acetylation, the RAW264.7 cells were transfected with SirT1 shRNA. Forty-eight hours after transfection, the cells were pretreated with salidroside for 2 h and then treated with LPS for additional 4 h. The SirT1 interference efficiencies were 58.62 and 60.87% through grey-level scanning and statistical analysis, respectively. As presented in Figure 8(c), the HMGB1 acetylation induced by LPS was reduced by pretreatment with salidroside in SirT1 shRNA-transfected cells. The results obtained from this study demonstrate that salidroside rescues the acetylation and nucleocytoplasmic translocation of HMGB1 induced by LPS in SirT1 shRNA-transfected cells by enhancing SirT1 expression.

## 4. Discussion

*Rhodiola rosea* has been widely used in traditional Chinese medicine with little toxicity. Salidroside, a bioactive compound isolated from *Rhodiola rosea*, possesses a variety of pharmacological effects, including anti-inflammatory [27], antitumour [28], and neuroprotection [29]. Our previous study demonstrated that salidroside attenuated LPS-induced inflammatory response by suppressing JAK2-STAT3 signalling pathway activation and preventing STAT3 transfer into the nucleus [25], but the detailed molecular mechanisms underlying the anti-inflammatory effect of salidroside remain elusive.

HMGB1, a ubiquitous DNA-binding protein, has been identified as a critical late mediator in endotoxaemia and sepsis [10]. The mechanisms of HMGB1 release require the translocation of HMGB1 from the nucleus to the cytoplasm and then the release into the extracellular space [13]. Recent studies have shown that posttranslational modifications of HMGB1, such as phosphorylation and acetylation, are involved in the translocation and subsequent secretion of HMGB1 [30, 31]. Whether salidroside affects the posttranslational modification and subsequent nucleocytoplasmic translocation of HMGB1 is still unclear. In this study, we first investigated the relationship between salidroside and HMGB1 release in vivo and in vitro and then explored the underlying mechanism.

In vivo, we detected the inhibitory effects of salidroside on HMGB1 release and lung injury by creating a rat model of sepsis. Administration of salidroside successfully inhibited the elevation of HMGB1 in CLP rats, and the congestion, oedema, and inflammatory cell infiltration induced by CLP were alleviated in the salidroside group (Figure 2). These results suggest that salidroside might act as an inhibitor of HMGB1 release. Nucleocytoplasmic translocation of HMGB1 is a key step for HMGB1 active release during inflammation. To clarify the effects of salidroside on HMGB1 nucleocytoplasmic translocation, RAW264.7 cells were pretreated with salidroside and then stimulated with LPS, resulting in the detection of HMGB1 release and translocation. The results presented in Figure 3 show that the levels of HMGB1 in cell culture supernatants were enhanced after LPS stimulation and peaked at approximately 12 h. Upon preretreatment with salidroside, LPS-induced HMGB1 release was inhibited in a dose-dependent manner. The levels of HMGB1 as well as its localization in the nucleus and cytoplasm showed that salidroside suppressed LPS-induced HMGB1 nucleocytoplasmic translocation (Figure 4).

Several posttranslational modifications affect the translocation of HMGB1 from the nucleus to the cytoplasm; for instance, LPS stimulation induces the phosphorylation and acetylation of HMGB1 and its subsequent secretion in RAW 264.7 cells [32, 33]. Consistent with these reports, our results showed that LPS clearly induced HMGB1 phosphorylation and acetylation and that pretreatment with salidroside reduced LPS-induced acetylation but not phosphorylation (Figure 5). Sirtuin 1 (SirT1), an NAD-dependent class III histone deacetylase, plays important roles in regulating HMGB1 acetylation [12, 33]. We subsequently investigated whether the effect of salidroside on HMGB1 acetylation was related to the expression of SirT1 and found that salidroside enhanced SirT1 expression in a dose- and time-dependent manner (Figures 6(a) and 6(b)). In addition, after treatment with LPS, SirT1 expression was attenuated compared with that of the control group in RAW264.7 cells. The results were consistent with those from a report by Ma et al. who found that LPS reduced the expression of SirT1 via p38 MAPK signalling [34]. However, salidroside pretreatment could reverse the reduction of SirT1 induced by LPS (Figure 6(c)). The AMPK-SirT1 signalling pathway regulates inflammatory signalling in various cells. To explore the possible signalling mechanism underlying salidroside regulation of SirT expression, the phosphorylation of AMPK was detected. As expected, salidroside could enhance AMPK phosphorylation and rescue the reduction of AMPK phosphorylation induced by LPS (Figures 6(d), 6(e), and 6(f)).

To confirm the roles of SirT1 in LPS-induced HMGB1 nucleocytoplasmic translocation and acetylation, RAW264.7 cells were transfected with Flag-SirT1 overexpression and control plasmids, and then, the nucleocytoplasmic translocation and acetylation of HMGB1 were detected. As expected, in control plasmid-transfected cells, LPS stimulation increased HMGB1 nucleocytoplasmic translocation and acetylation, whereas the nucleocytoplasmic translocation and acetylation of HMGB1 induced by LPS were clearly attenuated in Flag-SirT1 plasmid-transfected cells (Figure 7). To further verify the effects of salidroside on HMGB1 acetylation and nucleocytoplasmic translocation through increasing SirT1 expression, we also transfected SirT1 shRNA to first downregulate SirT1 expression and then treated the cells with salidroside to reverse the downregulation of SirT1. Our results in Figure 8 suggest that salidroside could rescue the acetylation and nucleocytoplasmic translocation of HMGB1 induced by LPS in SirT1 shRNA-transfected cells by upregulating the level of SirT1.

Recent studies have revealed that JAK-STAT1 signalling promotes HMGB1 acetylation and nucleocytoplasmic translocation [13]. This study suggests that JAK-STAT1 signalling is involved in the release of HMGB1 induced by LPS. In addition, it has been reported that ethyl pyruvate inhibits the acetylation and release of HMGB1 via SirT1/STAT1 signalling in LPS-activated RAW264.7 cells and peritoneal macrophages [33]. However, our previous study showed that salidroside did not inhibit LPS-activated STAT1 phosphorylation in RAW264.7 cells [25]. Hwang et al. reported that SirT1 interacts directly with HMGB1; however, upon stimulation with LPS or TNF-*α*, HMGB1 is acetylated and then the complex dissociates, leading to the release of HMGB1 [35]. Based on these results, we speculated that salidroside might reduce HMGB1 release through upregulating the AMPK-SirT1 signalling, inhibiting HMGB1 acetylation and then attenuating the dissociation of SirT1 and HMGB1. However, the effects of salidroside on the binding of SirT1 and HMGB1 require further investigation.

**Figure 9:**
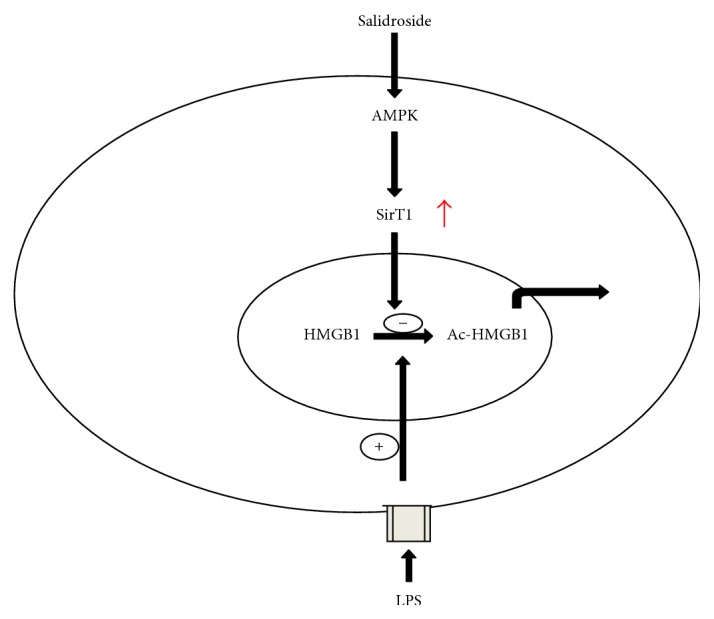
Schematic diagram illustrating the signalling pathways of salidroside inhibition of LPS-induced HMGB1 acetylation and nucleocytoplasmic translocation in RAW264.7 cells.

In conclusion, in the present study, salidroside was proven to inhibit HMGB1 release in LPS-induced RAW264.7 cells and reduce the serum HMGB1 level in CLP rats by suppressing LPS-induced HMGB1 acetylation, at least partially by regulating the AMPK-SirT1 signalling pathway (Figure 9). Our results suggest that salidroside may be considered a potential treatment for diseases in which HMGB1 is viewed as a target. This study also provides a new perspective for the anti-inflammatory function of salidroside.

## References

[1] Yamada S., Maruyama I. (2007). HMGB1, a novel inflammatory cytokine. *Clinica Chimica Acta*.

[2] Qin W. D., Mi S. H., Li C. (2015). Low shear stress induced HMGB1 translocation and release via PECAM-1/PARP-1 pathway to induce inflammation response. *PLoS One*.

[3] Bustin M. (1999). Regulation of DNA-dependent activities by the functional motifs of the high-mobility-group chromosomal proteins. *Molecular and Cellular Biololgy*.

[4] Lee D. U., Ko Y. S., Kim H. J., Chang K. C. (2016). 13-Ethylberberine reduces HMGB1 release through AMPK activation in LPS-activated RAW264.7 cells and protects endotoxemic mice from organ damage. *Biomedicine & Pharmacotherapy*.

[5] Dumitriu I. E., Baruah P., Valentinis B. (2005). Release of high mobility group box 1 by dendritic cells controls T cell activation via the receptor for advanced glycation end products. *The Journal of Immunology*.

[6] Scaffidi P., Misteli T., Fau-Bianchi M. E., Bianchi M. E. (2002). Release of chromatin protein HMGB1 by necrotic cells triggers inflammation. *Nature*.

[7] Youn J. H., Shin J. S. (2006). Nucleocytoplasmic shuttling of HMGB1 is regulated by phosphorylation that redirects it toward secretion. *The Journal of Immunology*.

[8] Yang H., Hreggvidsdottir H. S., Palmblad K. (2010). A critical cysteine is required for HMGB1 binding to Toll-like receptor 4 and activation of macrophage cytokine release. *Proceedings of the National Academy of Sciences of the United States of America*.

[9] Lei H., Wen Q., Li H. (2016). Paeonol inhibits lipopolysaccharide-induced HMGB1 translocation from the nucleus to the cytoplasm in RAW264.7 cells. *Inflammation*.

[10] Zhou H., Ji X., Wu Y. (2014). A dual-role of Gu-4 in suppressing HMGB1 secretion and blocking HMGB1 pro-inflammatory activity during inflammation. *PLoS One*.

[11] Bonaldi T., Talamo F., Scaffidi P. (2003). Monocytic cells hyperacetylate chromatin protein HMGB1 to redirect it towards secretion. *The EMBO Journal*.

[12] Zeng W., Shan W., Gao L. (2015). Inhibition of HMGB1 release via salvianolic acid B-mediated SIRT1 up-regulation protects rats against non-alcoholic fatty liver disease. *Scientific Reports*.

[13] Lu B., Antoine D. J., Kwan K. (2014). JAK-STAT1 signaling promotes HMGB1 hyperacetylation and nuclear translocation. *Proceedings of the National Academy of Sciences of the United States of America*.

[14] Zou J. Y., Crews F. T. (2014). Release of neuronal HMGB1 by ethanol through decreased HDAC activity activates brain neuroimmune. *PLoS One*.

[15] Xu W., Lu Y., Yao J. (2014). Novel role of resveratrol: suppression of high-mobility group protein box 1 nucleocytoplasmic translocation by the upregulation of sirtuin 1 in sepsis-induced liver injury. *Shock*.

[16] Xue B., Kahn B. B. (2006). AMPK integrates nutrient and hormonal signals to regulate food intake and energy balance through effects in the hypothalamus and peripheral tissues. *The Journal of Physiology*.

[17] Haigis M. C., Guarente L. P. (2006). Mammalian sirtuins—emerging roles in physiology, aging, and calorie restriction. *Genes & Development*.

[18] Fulco M., Cen Y., Zhao P. (2008). Glucose restriction inhibits skeletal myoblast differentiation by activating SIRT1 through AMPK-mediated regulation of Nampt. *Developmental Cell*.

[19] Yang Z., Kahn B. B., Shi H., Xue B. Z. (2010). Macrophage α1 AMP-activated protein kinase (α1AMPK) antagonizes fatty acid-induced inflammation through SIRT1. *The Journal of Biological Chemistry*.

[20] Tian Y., Ma J., Wang W. (2016). Resveratrol supplement inhibited the NF-κB inflammation pathway through activating AMPKα-SIRT1 pathway in mice with fatty liver. *Molecular and Cellular Biochemistry*.

[21] Sun P., Song S. Z., Jiang S. (2016). Salidroside regulates inflammatory response in Raw 264.7 macrophages via TLR4/TAK1 and ameliorates inflammation in alcohol binge drinking-induced liver injury. *Molecules*.

[22] Sun K. X., Xia H. W., Xia R. L. (2015). Anticancer effect of salidroside on colon cancer through inhibiting JAK2-STAT3 signaling pathway. *International Journal of Clinical & Experimental Pathology*.

[23] Wang J., Li J. Z., AX L., Zhang K. F., Li B. J. (2014). Anticancer effect of salidroside on A549 lung cancer cells through inhibition of oxidative stress and phospho-p38 expression. *Oncology Letters*.

[24] Si P. P., Zhen J. L., Cai Y. L., Wang W. J., Wang W. P. (2016). Salidroside protects against kainic acid-induced status epilepticus via suppressing oxidative stress. *Neuroscience Letters*.

[25] Qi Z., Qi S., Ling L., Lv J., Feng Z. (2016). Salidroside attenuates inflammatory response via suppressing JAK2-STAT3 pathway activation and preventing STAT3 transfer into nucleus. *International Immunopharmacology*.

[26] Xu S., Gao Y., Zhang Q. (2016). SIRT1/3 activation by resveratrol attenuates acute kidney injury in a septic rat model. *Oxidative Medicine and Cellular Longevity*.

[27] Li D., Fu Y., Zhang W. (2013). Salidroside attenuates inflammatory responses by suppressing nuclear factor-κB and mitogen activated protein kinases activation in lipopolysaccharide-induced mastitis in mice. *Inflammation Research*.

[28] Lv C., Huang Y., Liu Z. X., Yu D., Bai Z. M. (2016). Salidroside reduces renal cell carcinoma proliferation by inhibiting JAK2/STAT3 signaling. *Cancer Biomark*.

[29] Chen X., Zhang Q., Cheng Q., Ding F. (2009). Protective effect of salidroside against H_2_O_2_-induced cell apoptosis in primary culture of rat hippocampal neurons. *Molecular and Cellular Biochemistry*.

[30] Evankovich J., Sw C., Zhang R. (2010). High mobility group box 1 release from hepatocytes during ischemia and reperfusion injury is mediated by decreased histone deacetylase activity. *Journal of Biological Chemistry*.

[31] Taira J., Kida Y., Kuwano K., Higashimoto Y. (2013). Protein phosphatase 2A dephosphorylates phosphoserines in nucleocytoplasmic shuttling and secretion of high mobility group box 1. *Journal of Biochemistry*.

[32] Zhang X., Wheeler D., Tang Y. (2008). Calcium/calmodulin-dependent protein kinase (CaMK) IV mediates nucleocytoplasmic shuttling and release of HMGB1 during lipopolysaccharide stimulation of macrophages. *The Journal of Immunology*.

[33] Kim Y. M., Park E. J., Kim J. H., Park S. W., Kim H. J., Chang K. C. (2016). Ethyl pyruvate inhibits the acetylation and release of HMGB1 via effects on SIRT1/STAT signaling in LPS-activated RAW264.7 cells and peritoneal macrophages. *International Immunopharmacology*.

[34] Ma L., Zhao Y., Wang R. (2015). 3,5,4′-Tri-O-acetylresveratrol attenuates lipopolysaccharide-induced acute respiratory distress syndrome via MAPK/SIRT1 pathway. *Mediators of Inflammation*.

[35] Hwang J. S., Choi H. S., Ham S. A. (2015). Deacetylation-mediated interaction of SIRT1-HMGB1 improves survival in a mouse model of endotoxemia. *Scientific Reports*.

